# Adherence Behavior in Subjects on Hemodialysis Is Not a Clear Predictor of Posttransplantation Adherence

**DOI:** 10.1016/j.ekir.2019.04.028

**Published:** 2019-05-16

**Authors:** Abigail Hucker, Christopher Lawrence, Shivani Sharma, Ken Farrington

**Affiliations:** 1 Department of Psychology and Sport Sciences, School of Life and Medical Sciences, University of Hertfordshire, Hertfordshire, UK; 2 Renal Unit, Lister Hospital, Stevenage, Hertfordshire, UK; 3 Centre for Health Services and Clinical Research, University of Hertfordshire, Hertfordshire, UK

**Keywords:** adherence, hemodialysis, immunosuppression, kidney, phosphate, transplant

## Abstract

**Introduction:**

Nonadherence is common in both hemodialysis (HD) and kidney transplant recipients and is a major risk factor for poor clinical outcomes. This retrospective study explored whether nonadherent HD patients become nonadherent transplant recipients.

**Methods:**

Data were collected for 88 patients from the electronic patient system at a subregional renal unit about adherence to HD regimens in the 6 months before transplantation, and for 1 year posttransplantation following return transfer to the posttransplantation clinic from the transplanting center. Pretransplantation definitions of nonadherence included whether the patients: on average, shortened their dialysis prescription by >10 minutes; shortened it by >15 minutes; missed 2 or more HD sessions; and had mean serum phosphate levels >1.8mmol/l. Posttransplantation definitions of nonadherence included mean tacrolimus levels outside 5 to 10 ng/ml; and missed 1 or more posttransplantation clinic appointments.

**Results:**

Nonadherence ranged from 25% to 42% pretransplantation and from 15.9% to 22.7% posttransplantation, depending on how it was operationalized. There was little relationship between pretransplantation data and posttransplantation adherence, with the exception of a significant relationship between pretransplantation phosphate and posttransplantation clinic attendance. Patients who had missed 1 or more transplant clinic appointments had higher mean pretransplantation phosphate levels. Nonadherent patients with high phosphate levels pretransplantation and missed clinic appointments posttransplantation were significantly younger.

**Conclusion:**

Our findings provide little support for the likelihood of a strong direct relationship between pre and posttransplantation behaviors. The findings require confirmation and further research to assess whether interventions in relation to pretransplantation adherence may enhance adherence posttransplantation and improve outcomes.

Nonadherence is common in both HD patients and kidney transplant recipients. Hemodialysis regimens are complex and demanding, necessitating attendance at HD sessions, adherence to prescribed medications, and fluid and dietary restrictions.^1^, ^2^ Poor adherence can lead to poor clinical outcomes and increased risk of mortality,^3^, ^4^ as well as adding to health care costs.^5^, ^6^ There is a lack of consensus on definitions, which contributes to widely varying nonadherence rates in the HD population. Rates of skipping HD sessions vary between 0% and 32.3%, medication nonadherence between 1.2% and 81%, fluid restriction nonadherence from 3.4% to 74%, and nonadherence to dietary restrictions from 1.2% to 82.4%.^2^

There have been some attempts to achieve consensus. A United States Renal Data System (USRDS) study^3^ defined nonadherence in 4 ways: (i) skipping HD sessions; (ii) shortening HD sessions by 10 minutes or more; (iii) an interdialytic weight gain of more than 5.7% of patient dry weight; and (iv) serum phosphate of greater than 7.5 mg/dl (2.42 mmol/l). Using these definitions, the highest rates of nonadherence were found for shortening HD sessions (20.3%) and serum phosphate (22.1%). Applying the same criteria to data from the Dialysis Outcomes and Practice Patterns Study (DOPPS), levels of nonadherence across these 4 areas were 3.8%–19.6% for the overall international sample and 0.6%–20% for the European sample,^4^ and from 0.6%–23.8% for the overall European sample and 0.8%–21.9% for the UK sample.^7^

Compared with HD patients, kidney transplant recipients have improved quality of life, a less restrictive diet, longer life expectancy,^8^ and fewer psychological symptoms such as depression.^9^ Nonetheless, these patients are also required to make adjustments in their lifestyle such as adherence to immunosuppressants to prevent rejection, alongside attending clinic for regular check-ups and generally maintaining a good diet and activity level.^10^ The length of time that a patient waits for a kidney transplant varies in the UK across transplanting centers; however, the average wait is 2.5 to 3 years.^11^ For adult patients registered for a deceased donor transplant from April 2011 to March 2014, the median waiting time was 829 days and the median time from start of dialysis to kidney transplant from April 2016 to March 2017 was 1148 days.^12^

Nonadherence is a major risk factor for poor outcomes including graft survival.^13^ Reported rates of nonadherence to immunosuppressants in transplant patients vary, and again, definitions and methods of assessment are inconsistent. Methods such as self-report, electronic monitoring, reports from family, or health care professional observations are most common.^14^ Use of clinical data to assess nonadherence in this setting is less frequently used. A review of nonadherence to immunosuppressants^14^ reported nonadherence rates ranging from 2% to 67%, depending on definitions and methods deployed, with an average rate of 28% when adherence was measured by self-report.

It is important to consider how adherence behavior may transfer from 1 modality to another. For example, if poor adherence in HD patients pretransplantion could be identified and addressed, could posttransplantation adherence be improved and, with it, the risks of graft loss? Douglas *et al.*,^15^ conducted a longitudinal retrospective chart audit to examine this relationship, specifically examining pretransplantation adherence and posttransplantation outcomes in 126 renal transplant recipients. They defined nonadherence as having at least 1 chart note indicating pre- or posttransplantation nonadherence with a therapeutic regimen. Findings showed that 61% of patients identified as nonadherent before transplantation experienced graft loss or died. This research indicated a potential relationship between pretransplantation adherence and posttransplantation outcomes; however, the method of defining nonadherence was not particularly stringent.

More recently, Dobbels *et al.*^16^ prospectively followed 141 heart, liver and lung transplant recipients, examining pretransplantation predictors of posttransplantation outcomes. Independent predictors of nonadherence to immunosuppressants 1 year posttransplantation were pretransplantation nonadherence to taking medication, having less social support with medication taking, having higher education status, and having lower scores for the personality trait “conscientiousness.” A meta-analysis of 122 studies reporting associations between social support and patient adherence also suggested that poor social support is a key determinant of nonadherence to medical treatment regimens.^17^ In addition, pretransplant medication adherence was found to be the only predictor of late acute rejection. Although this research was not conducted in the kidney transplant population, it signals the importance of attending to pretransplantation behavioral patterns in predicting posttransplantation outcomes.

To our knowledge, there is little previous literature examining the relationship between clinical measures of pretransplantation adherence to HD and posttransplantation adherence in the renal transplant population. Although previous literature has highlighted predictors of nonadherence to HD and posttransplantation adherence separately, there is little exploration of whether there is a relationship that could help clinicians to identify aspects of pretransplantation nonadherence that act as potential risk factors for posttransplantation nonadherence. In addition, this could highlight patients who need to be targeted for intervention to address adherence concerns before transplantation to modify adherence behavior posttransplantation. It is clear from the literature that nonadherence both pretransplantation when on HD, and posttransplantation, are major risk factors for poor clinical outcomes and hence important to address. This retrospective study addresses whether nonadherent HD patients become nonadherent transplant recipients. It also considers whether there are particular patterns of nonadherence to HD that are more likely to associate with poor adherence after transplantation.

## Methods

This was a retrospective study carried out in a subregional renal unit. Data were collected from the electronic patient system about adherence to HD regimens in the 6 months before transplantation, and for 1 year posttransplantation after return transfer to the posttransplantation clinic from the transplanting center.

### Participants

The study population consisted of 88 adult (aged 18 years and more) kidney transplant recipients. Patients were eligible for inclusion if they had (i) received their transplant between 2006 and 2016, and (ii) had data available for a minimum of 6 months of HD before transplantation. We also included only those patients who were prescribed tacrolimus as their posttransplantation immunosuppressant, as this was the most common immunosuppressant across patients. Exclusion criteria included patients who were transferred later than 1 year posttransplantation back to the transplant clinic from the transplanting center. Further exclusions included patients who received HD for less than 6 months before transplantation, and patients who received home HD or peritoneal dialysis in the 6-month period before transplantation.

**Figure 1:**
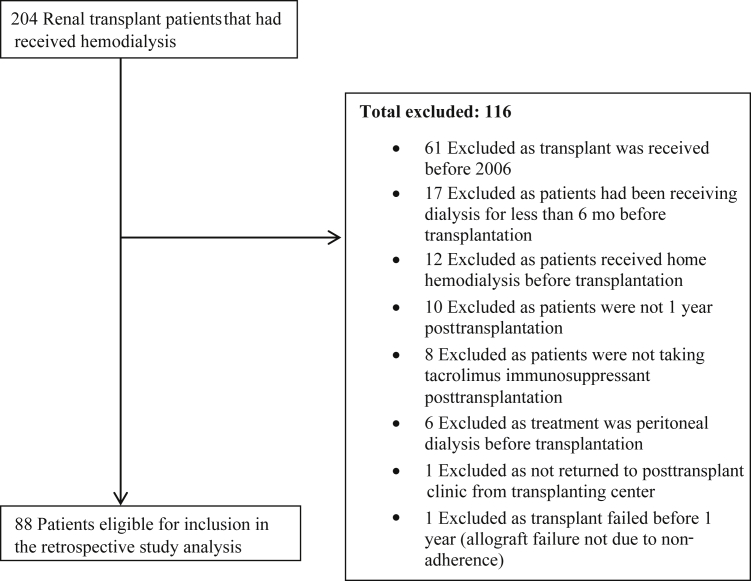
Flow diagram of participant study inclusion and exclusion.

Of a sample of 204 kidney transplant patients who had received HD as a treatment at some point before transplantation, 88 were eligible for inclusion in the study analysis. The remaining 116 patients were excluded. Figure 1 includes details of patient exclusions.

### Data Retrieved

Demographic data retrieved included age, sex, ethnicity, age at first dialysis session, age at transplantation, and Index of Multiple Deprivation (IMD) score from patient postcodes. Clinical data collected as part of routine care were retrieved that could provide indicators of nonadherence. Pretransplantation measures included the followiing: variance from dialysis prescription (in minutes of session length), missed number of dialysis sessions, dialysis vintage, residual kidney function (KRU), serum phosphate levels, parathyroid hormone (PTH), and interdialytic weight gain (IDWG). Missed dialysis due to hospitalization was not included as missed sessions. Means were calculated for the 6-month period before transplantation for pretransplantation measures, using measurements recorded as part of routine medical care (with the exception of missed dialysis sessions, which was a count). Posttransplantation measures included tacrolimus levels and their SD, number of missed clinic appointments, and donor type.

### Defining Adherence

There is no universally agreed way of defining adherence pre- and posttransplantation; therefore, based on previous literature^3^, ^4^, ^7^, ^18^ and clinical expected ranges, different cut-off points were applied to assess the data to explore potential relationships between pre- and posttransplantation adherence.

Pretransplantation definitions of nonadherence included patients: (i) on average, shortening their dialysis prescription by more than 10 minutes; (ii) on average, shortening their dialysis prescription by more than 15 minutes; (iii) missed 2 or more HD sessions; and (iv) had a mean serum phosphate level of 1.8 mmol/l or more.

Posttransplantation definitions of nonadherence included: (i) mean tacrolimus levels outside of the expected range within the first 2 years of 5 to 10 ng/ml after transplantation; and (ii) missing 1 or more posttransplantation clinic appointments.

For this study, a 6-month period was used to assess nonadherence pretransplantation, whereas previous studies^3^, ^4^, ^7^ defined nonadherence in a narrower time frame of 1 month. In addition to using shortening dialysis prescription by more than 10 minutes as used in previous research as a definition of nonadherence, more than 15 minutes was also used as a cut-off point, as this represented the top 25% of variance in dialysis prescription times for patients in this study. Previous studies used a higher nonadherence cut-off point for serum phosphate levels of 7.5 mg/dl (∼2.42 mmol/l). We chose to use a cut-off point in line with previous research using serum phosphate as a measure of nonadherence^19^ and in line with the recommended Renal Association serum phosphate levels of 1.1 to 1.7 mmol/l in the United Kingdom. Hence we use serum phosphate levels of 1.8 mmol/l or more to define nonadherence.^20^ Posttransplantation tacrolimus therapeutic range of 5 to 10 ng/ml was determined on the basis of clinical advice and previous research that used this range.^18^

### Statistical Analysis

As different markers were used to measure adherence pretransplantation compared to post- transplantation, a narrative comparison of the data is reported. Demographic data are reported for the study sample using means and frequencies. Comparisons of the data were completed using the McNemar test and Cochran Q for categorical data and *t* tests for continuous data. All tests were 2-tailed, and *P* values of less than 0.05 were considered to be significant. Logistic regressions were used to determine potential predictors of nonadherence both pre- and posttransplantation. The McNemar test was used to explore relationships between pre- and posttransplantation adherence. The data were analysed using SPSS version 25 (IBM SPSS, Armonk, NY).

### Approvals

This study was considered by the institutional review team at East and North Hertfordshire NHS Trust (RD2016-82) and was determined to be a service evaluation. Departmental agreement was provided for the service evaluation to be completed.

## Results

### Patient Characteristics

Of the 88 patients, 62.5% were male and 37.5% were female. Mean age at transplantation for the overall sample was 48.5 years (SD = 12.7), with no significant difference between sexes. The majority of patients (54.5%) were from white ethnic backgrounds, although a considerable proportion (45.5%) were also from other ethnic groups (Table 1).

**Table 1:** Demographic comparison of adherent and nonadherent patients pretransplantation

	n = 88 (%)	Pretransplantation shortening dialysis prescription >10 min		Pretransplantation shortening dialysis prescription >15 min		Pretransplantation missed dialysis sessions ≥2		Phosphate ≥1.8 mmol/l	
		Adherent	Nonadherent	Adherent	Nonadherent	Adherent	Nonadherent	Adherent	Nonadherent
		n = 53 (60.2%)	n = 35 (39.8%)	n = 66 (75%)	n = 22 (25%)	n = 64 (72.7%)	n = 24 (27.3%)	n = 51 (58%)	n = 37 (42%)
Age at transplant, mean (SD)	48.5 (12.7)	49.2 (13.4)	47.5 (11.6)	49.1 (13.1)	46.9 (11.4)	48.4 (12.8)	48.9 (12.6)	50.8 (11.7)	45.4 (13.4)^a^
Age at first dialysis, mean (SD)	44.9 (13.0)	45.3 (13.7)	44.3 (12.0)	45.1 (13.6)	44.2 (11.2)	44.7 (13.3)	45.5 (12.4)	47.2 (12.3)	41.7 (13.4)
Sex, n (%)									
Male	55 (62.5)	32 (60.4)	23 (65.7)	41 (62.1)	14 (63.6)	39 (60.9)	16 (66.7)	31 (60.8)	24 (64.9)
Female	33 (37.5)	21 (39.6)	12 (34.3)	25 (37.9)	8 (36.4)	25 (39.1)	8 (33.3)	20 (39.2)	13 (35.1)
Ethnicity, n (%)									
White	48 (54.5)	29 (54.7)	19 (54.3)	39 (59.1)	9 (40.9)	32 (50)	16 (66.7)	28 (54.9)	20 (54.1)
Nonwhite	40 (45.5)	24 (45.3)	16 (45.7)	27 (40.9)	13 (59.1)	32 (50)	8 (33.3)	23 (45.1)	17 (45.9)
Index of Multiple Deprivation, mean (SD)	5.5 (2.9)	5.3 (3.0)	5.9 (2.8)	5.6 (3.0)	5.2 (2.7)	5.3 (3.1)	5.9 (2.6)	6.0 (3.0)	4.8 (2.7)
Dialysis									
Dialysis vintage, mo Median (IQR)	26 (16, 49)	26 (16.3, 54)	26 (15, 40)	26 (17, 51)	24 (13, 39)	25 (16, 48)	27 (16, 50)	28 (15, 55)	24 (17, 40)
KRU median (IQR)	0.42 (0.01, 2.2)	0.30 (0.01, 1.2)	1.28 (0.07, 2.7)^a^	0.41 (0.01, 1.9)	0.64 (0.01, 2.6)	0.39 (0.01, 2.2)	0.81 (0.01, 2.3)	1.1 (0.01, 2.8)	0.10 (0.01, 0.86)^a^
PTH median (IQR)	37 (25, 57)	35 (22.2, 57)	43 (25, 59)	36 (25, 57)	43 (25, 60)	38 (26, 57)	30 (14, 70)	30 (17, 51)	50 (34, 72)^a^
IDWG median (IQR)	1.79 (1.24, 2.4)	1.8 (1.4, 2.4)	1.8 (1.1, 2.4)	1.8 (1.2, 2.4)	1.8 (1.1, 2.5)	1.8 (1.2, 2.4)	1.9 (1.3, 2.5)	1.7 (.94, 2.4)	1.8 (1.6, 2.5)

IDWG, interdialytic weight gain (kg); IQR, interquartile range; KRU, urea clearance (ml/min); PTH, parathyroid hormone (pmol/l).

a *P* < 0.05.

Indices of deprivation rank every postcode in England from 1 (most deprived) to 32, 844 (least deprived). These are split into deciles of 1 to 10 from most deprived to least deprived, dividing them into 10 equal groups, ranging from 1 = from the most deprived 10%, to 10 = the least deprived 10%. Across the overall sample, 23.9% (n = 21) of patients lived in neighborhoods that fall in the 20% most deprived small areas in England. Findings differed across ethnicity, with 40% (n = 16) of nonwhite patients shown to live in neighborhoods that fall in the 20% most deprived small areas in England as compared to only 10.4% (n = 5) of white patients. Chronic glomerulonephritis (21.6%: n = 19), diabetic nephropathy (20.5%: n = 18), polycystic kidney disease (13.6%: n = 12), chronic pyelonephritis (9.1%, n = 8), and hypertension (4.5%: n = 4) accounted for the majority of cases. Etiology was uncertain for more than a quarter of the sample (28.4%, n = 25).

### Clinical Pretransplantation Data

Nonadherence ranged from 25% to 42%, depending on how it was operationalized. There were no significant demographic differences between groups across measures, with the exception being that patients categorized as nonadherent based on phosphate levels were significantly younger at transplantation (t[86] = 1.99, *P* = 0.049) than those categorized as adherent (Table 1). Cochran Q was used to determine whether there were any differences in patients identified as nonadherent across the 4 pretransplantation measures. There was a statistically significant difference in the proportion of nonadherent patients across the 4 nonadherence measures (χ^2^[3] = 9.79, *P* = 0.020).

We explored whether pretransplantation clinical data predicted pretransplantation adherence. Dialysis vintage, KRU, serum phosphate, PTH, and IDWG were compared independently across adherent and nonadherent patients using the 4 pretransplantation adherence measures. All measures were skewed with the exception of serum phosphate. There was a significant difference for KRU between adherent and nonadherent patients, when adherence was defined as shortening dialysis by more than 10 minutes (U = 684.5, *P* = 0.035), with adherent patients having lower residual kidney function than nonadherent patients (Table 1). No other significant differences were observed between adherent and nonadherent patients when adherence was defined as shortening dialysis by more than 10 minutes or by more than 15 minutes. Significant differences were observed when adherence was defined using serum phosphate levels. Patients who were categorized as nonadherent based on their phosphate levels had lower KRU (U = 648, *P* = 0.011), and higher parathyroid hormone levels (U = 545.5, *P* = 0.002) (Table 1).

Logistic regression analyses were conducted to identify possible predictors of nonadherence among pretransplantation patients. Factors included in the models were age at transplantation, sex, ethnicity, Index of Multiple Deprivation score, and dialysis vintage. No significant predictors of nonadherence were identified for any of the nonadherence measures.

### Clinical Posttransplantation Data

Patients with mean tacrolimus levels outside the range expected within the first 2 years of 5 to 10 ng/l were highlighted. Of the 88 patients, 14 (15.9%) had tacrolimus levels outside the expected range of 5 to 10 ng/l. Ten patients were male and 4 were female. There were equal numbers of white and nonwhite patients (n = 7). There were no significant demographic or clinical differences between adherent and nonadherent patients defined in this way.

When nonadherence was defined using the number of missed posttransplantation clinic appointments as 1 or more, 20 patients (22.7%) were identified as nonadherent. No significant demographic differences were observed between groups when posttransplantation adherence was defined in this way, except that nonadherent patients were significantly younger when they underwent transplantation (t[86] = 2.14, *P* = 0.035) and significantly younger when starting dialysis (t[86] = 2.07, *P* = .041), than those categorized as adherent (Table 2).

**Table 2:** Demographic comparison of adherent and nonadherent patients posttransplantation

	Posttransplantation tacrolimus levels		Posttransplantation missed clinic appointments ≥1	
	Adherent	Nonadherent	Adherent	Nonadherent
	n = 74 (84.1%)	n = 14 (15.9%)	n = 68 (77.3%)	n = 20 (22.7%)
Age at transplant, mean (SD)	48.8 (12.9)	46.8 (11.7)	50.0 (12.1)	43.3 (13.4)^a^
Age at first dialysis, mean (SD)	45.1 (13.3)	43.50 (11.7)	46.4 (12.2)	39.7 (14.6)^a^
Dialysis vintage, mo, mean (SD)	35.9 (27.6)	29.8 (16.7)	35.4 (26.4)	33.1 (26.2)
Gender, n (%)				
Male	45 (60.8)	10 (71.4)	42 (61.8)	13 (65.0)
Female	29 (39.2)	4 (28.6)	26 (38.2)	7 (35.0)
Ethnicity, n (%)				
White	41 (55.4)	7 (50)	37 (54.4)	11 (55.0)
Nonwhite	33 (44.6)	7 (50)	31 (45.6)	9 (45.0)
Index of Multiple Deprivation, mean (SD)	5.7 (2.9)	4.6 (2.9)	5.69 (3.1)	4.9 (2.4)

a *P* < 0.05.

Logistic regression analyses were conducted to identify possible predictors of nonadherence among posttransplantation patients in this study. No significant predictors for nonadherence to tacrolimus immunosuppressant medication were identified. Phosphate levels of 1.8mmol/l or more pretransplantation were identified as predicting higher odds of nonattendance at posttransplantation clinic appointments (Table 3).

**Table 3:** Predictors of nonadherence posttransplantation

	Odds ratio (95% confidence interval) by nonadherence measure	
	Posttransplantation tacrolimus levels	Posttransplantation missed clinic appointments ≥1
Age at transplant, yr	0.99 (0.93, 1.05)	0.98 (0.94, 1.04)
Male vs. female	2.09 (0.49, 8.88)	0.80 (0.23, 2.86)
White vs. nonwhite	0.89 (0.23, 3.46)	1.40 (0.34, 5.82)
Index of Multiple Deprivation	0.85 (0.66, 1.09)	0.89 (0.70, 1.14)
Dialysis vintage, mo	0.99 (0.96, 1.02)	1.00 (0.97, 1.02)
Variance in dialysis time from prescription, min	1.01 (0.97, 1.04)	1.05 (0.99, 1.11)
Missed dialysis sessions >2 vs. <2	0.86 (0.19, 3.84)	0.60 (0.14, 2.58)
Phosphate ≥1.8 mmol/l vs. <1.8 mmol/l	0.69 (0.17, 2.80)	4.19 (1.15, 15.24)^a^
Donor type deceased vs. living	0.28 (0.06, 1.19)	2.42 (0.41, 14.29)

a *P* < 0.05.

In addition to looking at tacrolimus using the mean levels, the SD and coefficient of variation (CV) was also calculated to examine the variation in tacrolimus levels for each patient for the 1-year period recorded posttransplantation. A nonadherence cut-off point of SD of greater than 2.0 was used in line with previous research,^21^ and a tacrolimus CV% cut-off point of 41% was used, again in line with previous research.^22^ Logistic regression analyses were conducted to identify potential predictors of these parameters. No significant predictors were identified. There was no difference between the proportion of nonadherent patients defined in terms of highly variable tacrolimus levels (tacrolimus CV% > 41%) and defined in terms of missed clinic appointments (*P* = 0.47 by McNemar test).

### Comparing Pre- and Posttransplantation Adherence

In general, the prevalence of nonadherence was greater pretransplantation than posttransplantation. The prevalence of pretransplantation nonadherence defined by shortened dialysis by more than 10 minutes was greater than posttransplantation nonadherence adherence defined by both tacrolimus levels and by missed posttransplantation clinic appointments (*P* = 0.001 and 0.029, respectively; McNemar test). Likewise, the prevalence of pretransplantation nonadherence determined by phosphate levels was greater than the prevalence of posttransplantation nonadherence determined by both tacrolimus levels (*P* < 0.001) and by missed posttransplantation clinic appointments (*P* = 0.003). No other significant differences were found when comparing pre- and posttransplantation groups (Table 4).

**Table 4:** Comparing pretransplantation adherence to posttransplantation adherence measures

	Pretransplantation shortening dialysis prescription >10 min		*P*	Pretransplantation shortening dialysis prescription >15 min		*P*	Pretransplantation missed dialysis sessions ≥2		*P*	Phosphate ≥1.8 mmol/l		*P*
	Adherent	Nonadherent		Adherent	Nonadherent		Adherent	Nonadherent		Adherent	Nonadherent	
	n = 53 (60.2%)	n = 35 (39.8%)		n = 66 (75%)	n = 22 (25%)		n = 64 (72.7%)	n = 24 (27.3%)		n = 51 (58%)	n = 37 (42%)	
Posttransplantation determined by tacrolimus levels												
Adherent, n = 74	45	29	.001^a^	55	19	0.20	53	21	0.11	43	31	<.001^a^
Nonadherent, n = 14	8	6		11	3		11	3		8	6	
Posttransplantation missed clinic appointments ≥1												
Adherent, n = 68	40	28	0.029^a^	48	20	0.87	49	19	0.61	45	23	0.003^a^
Nonadherent, n = 20	13	7		18	2		15	5		6	14	

a *P* < 0.05.

We explored the relationship between pretransplantation demographic and clinical data to posttransplantation adherence. Of the 28 patients categorized as nonadherent to either one (n = 22) or both (n = 6) posttransplantation measures, 46.4% (n = 13) were nonadherent to 2 or more pretransplantation measures, compared with 32.2% (n = 9) who were nonadherent on a single pretransplantation measure. The remaining 21.4% (n = 6) of nonadherent patients posttransplantation were adherent to all pretransplantation measures. In general, there was only a weak relationship between pretransplantation data and posttransplantation adherence. The exception was that patients who had missed 1 or more posttransplantation clinic appointments had higher mean pretransplantation phosphate levels (mean = 1.92, SD = 0.41) compared with those who had missed none (mean = 1.69, SD = 0.40; t[86] = 2.25, *P* = 0.027). This finding suggests that patients with higher phosphate levels pretransplantation are more likely to miss clinic appointments posttransplantation. There was no relationship of interdialytic weight gain with posttransplantation adherence, even when the analysis was confined to patients with no residual kidney function pretransplantation.

## Discussion

The primary aim of this single-center retrospective study was to explore whether patterns of adherence behavior in patients on HD relate to posttransplantation adherence. Our findings do not support the likelihood of a strong direct relationship between these behaviors. However, the possibility remains of some overlap of nonadherent behaviors in these 2 settings, as evidenced by our finding of a relationship between pretransplantation phosphate control and subsequent attendance at posttransplantation follow-up.

The number of patients categorized as nonadherent was dependent on how nonadherence was defined. Pretransplantation nonadherence ranged from 25% to 42%, and posttransplantation nonadherence ranged from 15.9% to 22.7%, depending on definition. Our finding of a higher nonadherence rate for phosphate control than that quoted in the literature^3^, ^4^ is highly likely to be due to our using a lower cut-off point for nonadherence, in line with previous research from our unit^19^ and UK clinical practice guidelines.^20^

Defining pretransplantation adherence in terms of phosphate control, nonadherent patients were found to be of younger age at transplantation, to have less residual kidney function, and to have higher PTH levels. Both latter factors have well-established effects on phosphate control. Previous studies have also demonstrated negative correlations between age and phosphate control. Longer dialysis vintage was also associated with lower phosphate levels.^2^ Nevertheless, our findings may help to define a group of patients in whom targeted intervention may improve aspects of adherence pretransplantation and potentially posttransplantation.

Posttransplantation predictors of nonadherence, defined in terms of number of missed clinic appointments, similarly indicated that nonadherent patients were significantly younger at transplantation and at dialysis initiation. These patients also had higher phosphate levels pretransplantation, above the Renal Association−recommended range, indicating inadequate phosphate control. Previous literature using serum phosphate as a clinical measure of nonadherence also indicated phosphate control as a major issue for HD patients.^19^, ^23^ This is similar to our findings, which showed a mean phosphate level of 1.74 (SD = 0.41) for the overall sample. Logistic regression identified that phosphate levels of 1.8 mmol/l or more predicted higher odds of nonattendance at posttransplantation clinic appointments.

However, serum phosphate levels are affected by clinical variables and diet, and therefore their reliability as a measure of nonadherence should be interpreted with caution.^23^ There are multiple factors that could influence adherence to phosphate treatment, such as complex treatment regimen, high pill burden, side effects, and lack of immediate symptomatic benefit.^23^ It has been suggested that dietary and fluid restrictions may require more patient willpower in order to adhere. A study found that 57.6% of patients reported difficulty adhering to dietary prescription, and 56.3% reported that this was due to an inability to resist favorite foods.^2^ In addition, in the same study, 62% of patients reported some difficulty adhering to fluid restrictions, and 43.7% were unable to control their desire for fluid. This suggests that these aspects of the treatment regimen may be more challenging to adhere to and could explain why nonadherence rates are higher for these measures of pretransplantation nonadherence. This suggests that phosphate may be a better indicator of pretransplantation nonadherence than other measures because of the multifaceted nature of the behaviors required to manage phosphate levels, which encompass dietary restriction, phosphate binder medication adherence, and adherence to dialysis protocols. Age may be a factor in this relationship, as nonadherent patients judged by this parameter pretransplantation were significantly younger, as were those who missed posttransplantation clinic appointments. On the other hand, the absence of other significant predictors of posttransplantation nonadherence increases the possibility that this association was a chance finding.

We found that the prevalence of nonadherence was greater pretransplantation by some but not all measures. This may suggest that adherence with treatment posttransplantation is more manageable than adherence to treatment pretransplantation, which involves both HD and associated medications. However, the measures of adherence used in these setting are, of necessity, very different, so this interpretation needs to be treated with caution.

Overall, these findings suggest that pre- and posttransplantation adherence are only weakly associated. The relationship is complex. The challenges that patients experience with adherence pretransplantation may be different posttransplantation. There are multiple potential factors. For example, whether a patient has received a living or deceased donor organ may play a role in behavior modification.^24^, ^25^ In addition, anxiety, depression,^9^, ^26^ and socio-economic factors^12^ have been associated with nonadherence. Pretransplantation, patients on HD are usually entitled to free prescriptions. However, although posttransplantation clinic appointments are covered via the National Health Service (NHS), posttransplantation medication is not covered (unless patients meet the criteria for prescription payment exemption). This additionally could contribute to differences in nonadherence rates. Our findings do support existing literature pertaining to adherence in specific renal replacement therapy (RRT) modalities, for example, the relationship between younger age and nonadherence.

This study has both strengths and limitations. Although our study suggests the possibility of a complex relationship between pre- and posttransplantation adherence, the findings should be interpreted with caution. The sample size was small and single-centered. Younger age predicted high phosphate levels pretransplantation. We found no other major associations. However, the time frame in which nonadherence was assessed pretransplantation was 1 month in the previous literature,^3^, ^4^, ^7^ whereas this study assessed nonadherence over the 6 months pretransplantation. Our 6-month assessment of nonadherence may provide a more stable picture of patient behavioral patterns. In addition, a follow-up of 1 year may be too short a period for exploring posttransplantation nonadherence, as research suggests that rates of nonadherence can increase as time from transplantation increases.^27^ Finally, although we have attempted to delineate clinically relevant indices of nonadherence in the transplant population, there may be other parameters that may be more relevant. This could indicate the need to identify clinically relevant definitions that accurately measure nonadherence rates so as to ensure that this is reported reliably in future research. Notwithstanding these limitations, our study is 1 of the few to consider how patterns of adherence vary within patient groups as they transition between RRT modalities.

## Conclusion

Poor phosphate control pretransplantation was associated with some aspects of adherence posttransplantation. However, our findings do not indicate a strong direct relationship between pre- and posttransplantation adherence. Whatever measure of adherence used pretransplantation, nonadherence is less posttransplantation and, in some cases, significantly so. However, the only adherence parameter that predicted posttransplantation adherence was pretransplantation phosphate control. Although some patients do improve adherence to treatment posttransplantation, nonadherence remains an issue for a proportion of patients posttransplantation. Nonadherent patients pretransplantation should be reviewed on a case-by-case basis for transplant eligibility, to determine whether adherence behavior could change posttransplantation or whether interventions are needed pretransplantation before wait listing. These findings require confirmation and further work to assess whether interventions in relation to pretransplantation adherence may enhance adherence posttransplantation and hence improve outcomes. Furthermore, enhancing patient understanding about the importance of medication and engaging in treatment regimens could help to improve adherence posttransplantation.

## Disclosure

All the authors declared no competing interests.

## Author Contributions

KF conceived the study concept. All authors (AH, CL, SS, KF) contributed to the design of the study. KF and CL provided expertise in nephrology, including hemodialysis and renal transplant populations. AH conducted the evaluation. AH retrieved and analyzed the data and wrote the first draft of the manuscript. All authors provided feedback during the development of the manuscript and approved the final manuscript.
